# Insects as Food: Consumers’ Acceptance and Marketing

**DOI:** 10.3390/foods12040886

**Published:** 2023-02-19

**Authors:** Asmaa Alhujaili, Giuseppe Nocella, Anna Macready

**Affiliations:** 1 Department of Applied Economics and Marketing, School of Agriculture Policy and Development, University of Reading, Reading RG6 6AR, UK; 2 Department of Agribusiness and Consumer Science, School of Agricultural and Food Sciences, King Faisal University, Hofuf 31982, Saudi Arabia

**Keywords:** alternative protein, entomophagy, marketing mix, psychological factors, systematic review

## Abstract

The growing demand for livestock products is associated with an increase in environmental, economic, and ethical issues. New alternative sources of protein such as edible insects have recently been developed to tackle these issues with fewer drawbacks. However, several challenges are associated with insect-based food, mainly regarding consumer acceptance and commercialization. In this systematic review, we explored these challenges by reviewing 85 papers from 2010 to 2020, which were selected following the PRISMA methodology. Additionally, we applied the SPIDER (Sample, Phenomenon of Interest, Design, Evaluation, and Research type) tool for developing the inclusion criteria. Our analysis adds new knowledge to previous systematic reviews on this topic. It reveals both a comprehensive framework of factors influencing consumers’ acceptance of insects as food and aspects of the marketing mix of these products. Disgust, food neophobia, familiarity, visibility of insects, and taste appear to be the most significant factors that can prevent consumers from consuming insects as food. The motivations for acceptance are found to be familiarity and exposure. The results of this review provide insights for policymakers and stakeholders who wish to develop marketing strategies that can increase consumer acceptance of insects as food.

## 1. Introduction

The dramatic increase in food insecurity is globally affecting around 2 billion people, with the COVID-19 pandemic projected to add 83–132 million individuals [1]. Food insecurity will also be exacerbated by the growth in the world population, which is projected to rise from 7.7 billion in 2019 to 9.7 billion in 2050 [2]. These issues are accompanied by an increase in the demand for livestock production, which is associated with environmental, economic, and ethical problems [1,3,4,5,6]. Therefore, identifying alternative sources of protein can be helpful for policymakers and stakeholders tackling these issues. Offsetting the predicted food shortages and mitigating the consequences of increased food production by using meat alternatives will be critical to reducing negative consequences for human beings and the environment. Some of these alternatives exist in the global market (e.g., plant-based meat), others exist in more specialized markets (e.g., insect-based food), while the rest are not yet commercially available (e.g., cultivated meat).

In the world, about 2 billion people consume insects, namely over 2000 species of edible insects, and the majority of these people are located in developing countries [7,8,9]. In Africa, in countries such as the Democratic Republic of the Congo, Congo, the Central African Republic, Cameroon, Uganda, Zambia, Zimbabwe, Nigeria, and South Africa, the most commonly eaten insects include caterpillars, termites, crickets, and palm weevils. Entomophagy is also a common practice in Asia and local communities of South America.

Insects, due to their high protein, healthy fat, calcium, iron, and zinc content, are considered a promising alternative to protein obtained from farm animals [10,11]. Unlike plants, they provide food with complete animal protein [12]. Production of insects such as mealworms releases less greenhouse gas (GHG) emissions, and their use requires fewer natural resources (e.g., water, land, energy) than conventional meat protein sources [13,14]. Moreover, insects are highly efficient at converting their own food intake into protein. For example, crickets’ feed-conversion ratio is twice that of chickens, four times that of pigs, and twelve times that of cattle [15]. Thus, if livestock production is partially replaced by insect production, more land and grain for livestock feed would be available for crop production and human consumption, respectively. However, the sustainability of insects as food depends on many factors, such as the species used, the type of feed required, and the energy consumed when producing insect-based products [16]. In addition, as with other novel foods, insect production requires the development of new value chains and attention to issues such as production costs, food safety, and consumer acceptance [15,17,18].

Interest in exploring consumer acceptance of insects as food has increased rapidly in the past decade [19]. We identified four systematic reviews of this topic with different focuses, aims, and criteria [20,21,22,23]. The previous systematic reviews have targeted developed countries, with no consistent framework used in reporting the results. Exceptionally, Onwezen et al. [23] adopted Siegrist’s [24] framework for consumer acceptance of novel food.

To the best of our knowledge, only two specific frameworks have been developed to analyze the factors that influence consumer acceptance of entomophagy [25,26]. The Lensvelt and Steenbekkers framework [25] for consumer acceptance of entomophagy identified three categories: (1) product attributes (e.g., price, quality, health benefits/risks, naturalness, and convenience); (2) trust and social norms; and (3) psychological factors (attitudes and culture). Kauppi et al. [26] identified two categories: consumer factors and the product’s commercial potential.

To add value to previous systematics reviews, the following questions were explored: Would the factors influencing the acceptance of insect-based food identified from previous frameworks be reshaped? Do these factors influence consumers in developed and developing countries in the same way? Which marketing strategies might best benefit retailers and food industries to increase consumer acceptance of these products?

## 2. Materials and Methods

### 2.1. Identification of the Relevant Articles

In August 2021, a literature search was conducted in selected bibliographic databases to collect information from 2010–2020 necessary to answer our research questions. Bibliographic databases are broadly defined as digital collections of references to published sources (e.g., journal articles, books, and conference proceedings) tagged with specific titles, author names, affiliations, and abstracts [27]. We performed a search in three major multidisciplinary bibliographic databases (*ScienceDirect*, *Web of Science,* and *Scopus*), combining Boolean operators (AND, OR, and NOT) and key words identified by researchers involved in this study. The main keywords relating to the product included “Insect”, “edible”, “entomophagy”. Keywords relating to the consumer included “acceptance, “preferences”, “perception”, “values”, “attitudes”, “reaction”, “knowledge”, “behavio*”, “consumption”, “liking”, and “intention”. We also searched for “willingness to...”, “adopt”, “purchase”, “pay”, “buy”, “try”, “eat”, and “consume”.

**Figure 1:**
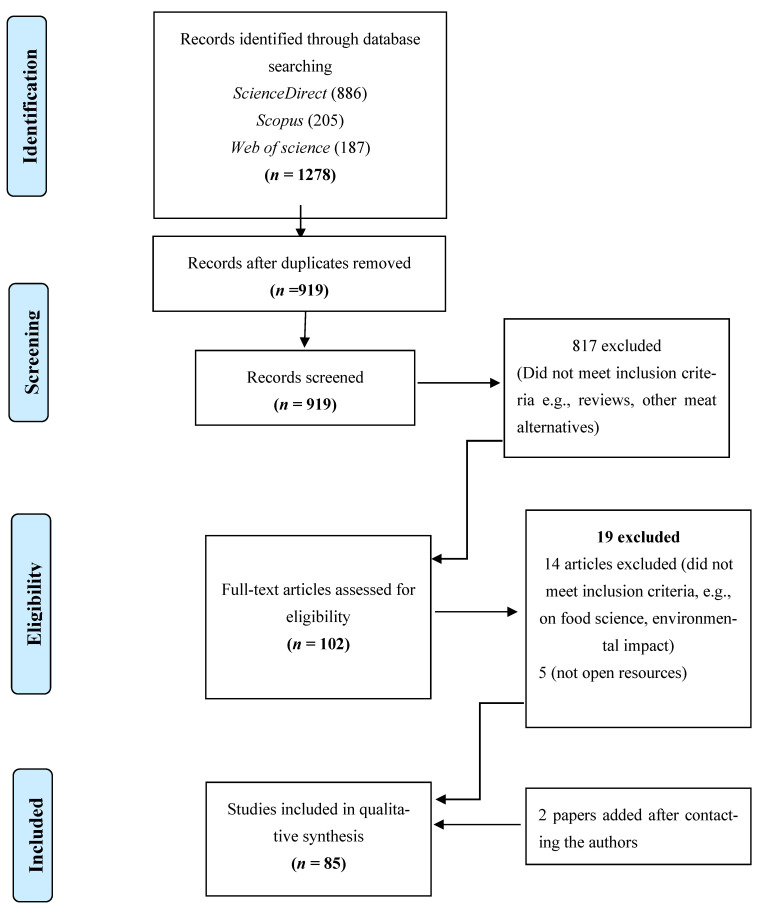
Flow chart of the systematic review of insects as food.

To improve the reporting of this systematic review, we followed the Preferred Reporting Items for Systematic Reviews and Meta-Analyses (PRISMA) statement [28] as illustrated in Figure 1. Steps 1 and 2 were developed by exploring the databases, while steps 3 and 4 implemented the Sample, Phenomenon of Interest, Design, Evaluation, and Research type (SPIDER) tool, which is recommended for answering research questions from both qualitative and mixed-methods research studies [29]. The English language was selected to identify original papers researching insects as food in consumer studies published in peer-reviewed journals from 2010–2020. We applied the SPIDER tool to categorize the inclusion/exclusion criteria as indicated in Table 1.

In total, 1278 articles were selected: 886 from *ScienceDirect*, 205 from *Scopus*, and 187 from *Web of Science*. A total of 359 duplicate articles were eliminated using Mendeley, and 817 further articles did not meet the inclusion criteria. Nineteen articles were excluded because they did not answer research questions, and where the study’s details were unclear, authors were contacted, with two articles subsequently included. Overall, 85 studies were included in the final sample.

### 2.2. Data Extraction Process

Data from the reviewed papers were extracted and checked by two reviewers; disparity was resolved by discussion and consensus. Information extracted from studies included in this systematic review has highlighted aspects such as the country in which the research was conducted, sample characteristics, methods used, product details, insect type, and main findings, as illustrated in Table S1 and Table S2.

## 3. Results

### 3.1. Overview of Studies Included in This Review

**Figure 2:**
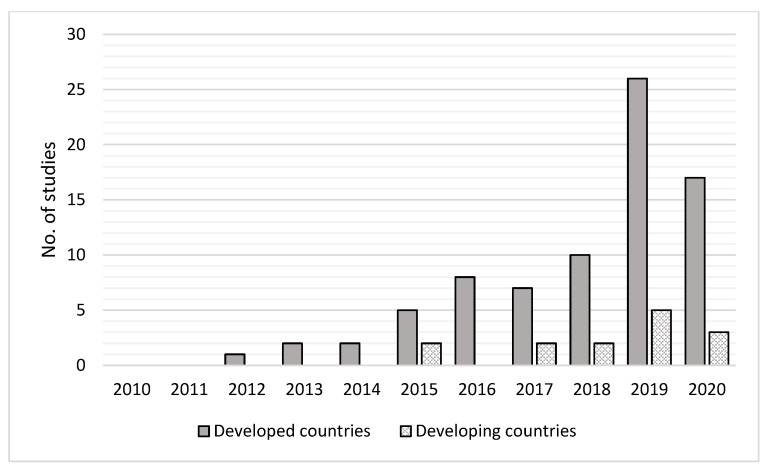
Comparison of the number of studies conducted in developed and developing countries.

Four main observations emerged from the analysis of the included studies. First, 85 studies were conducted in 32 countries, of which, according to the development status identification of the United Nations classification 2022, 22 are developed and 10 developing. There were 78 studies that referred to at least one developed country, and only 14 investigated this topic in a developing country. For developed countries, there was a significant upward trend over the years in the number of studies conducted. This is possibly due to the growing interest of researchers, food industries, and policy makers in insects as an alternative source of protein to provide solutions for food insecurity and the unsustainability of meat production (Figure 2). Furthermore, possible reasons for the discrepancy in the number of studies conducted in developed and developing countries could be identified by their disparity of economic resources and by the fact that in some developing countries, people already consume insects. The decreased number of studies observed in 2020 may have been caused by the COVID-19 pandemic.

Second, 79 studies used quantitative techniques, and 10 used qualitative methods such as in-depth interviews and focus groups, with some studies using both techniques. These studies varied in reporting the results in relation to the sample as many of them did not report the effect of size, education level, age, and gender. Where age was reported, we noted a bias towards younger participants, with an average age of 37.41 (s = 9.12) across all studies. This may be due to the online nature of the studies but also to the difficulty of interviewing older people. The average age for participants in experiments was 27.48 (s = 8.21). These were only conducted in developed countries, and many involved university students e.g., [30,31]. Women were better represented than men in both surveys and experiments (55 and 53 countries, respectively), which is interesting because the literature suggests that females’ acceptance of insects as food is lower than males’ [21,22,23]; thereby, we would expect them to be less willing to participate in studies involving insects as food.

Third, many studies explored consumer acceptance of insects as food in general without mentioning the types of insects (e.g., [32,33,34,35]), which could lead to misinterpretation of consumers’ acceptance [20]. When the insect type was specified, the most investigated were crickets in 36 studies, e.g., [36,37,38]; followed by mealworms in 35 studies, e.g., [39,40]; and grasshoppers in 15 studies, e.g., [41,42,43]. The popularity of these species may be because they are already used as feed for pets [19], they are more currently available in Western markets [36,44,45,46], and are the most likely growth markets in the future. Two studies included spiders and scorpions, which belong to the subphylum of arachnids. Even if arachnids belong to the phylum of arthropod like insects, they are not the same, but they are often considered to be edible [37].

Fourth, while many researchers did not specify the product details, most of those who did specify them considered insects when they were invisible in the products being discussed. This could be because Western consumers may be more willing to eat insects when they are invisible and highly processed in the food [16]. The most thoroughly investigated insect-based products were burger patties in 20 studies, e.g., [47,48]; protein bars in 15 studies, e.g., [44,49]; and cookies in 8 studies, e.g., [50,51]. Where insects were visible in the product, they were mostly described as fried in 10 studies, e.g., [40,52]; whole in 7 studies, e.g., [53,54]; or dried in 5 studies, e.g., [55,56]. 

**Figure 3:**
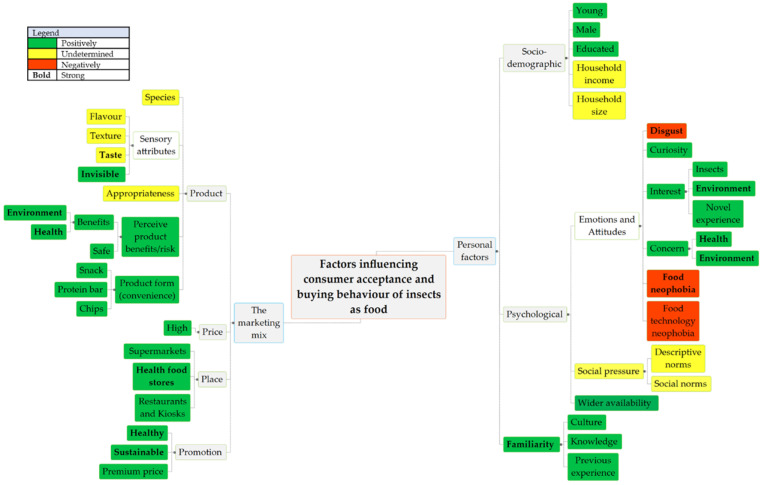
Factors influencing consumer acceptance and buying behavior of insects as food.

Results emerging from this systematic review are predominantly concerned with consumer acceptance of insects as food in Western and developed countries. This indicates a need for more studies in developing countries because the increase in livestock demand will make meat production in these countries unsustainable; therefore, exploring meat alternatives can contribute to mitigating this problem [57,58]. Due to the lack of consistent reporting of participants and product characteristics, our ability to form reliable general conclusions is limited and highlights the need for greater precision in the construction, administration, and reporting of future studies in this area. Bearing this in mind, Figure 3 illustrates that the factors influencing consumer acceptance and buying behavior of insect food can be classified as (i) personal factors (socio-demographic, psychological, and familiarity) and (ii) the elements of the marketing mix (product, price, promotion, and place). Factors highlighted in green and red can influence consumer acceptance positively and negatively respectively, while the influence of those highlighted in yellow is undetermined. Furthermore, when the identified factors appear in bold, their influence on consumer acceptance of insect food, concerning both personal factors and the elements of the marketing mix, is strong.

### 3.2. Personal Factors

Several personal factors appeared to be significant predictors of consumer acceptance of insects as food.

#### 3.2.1. Sociodemographic Characteristics

Results of this systematic review show that gender was the most investigated consumer characteristic, followed by age, level of education, household income, and family size, with comparisons being made between different regions of the same countries. However, for developing countries, only five studies reported information about gender and age.

The majority of studies found that gender is a significant predictor, with males more willing than females to accept insects in various products regardless of the visibility of insects in the food [32,37,40,44,51,52,59,60,61,62,63,64,65,66,67,68,69,70,71]. For instance, an online survey conducted in Italy found that males were 2.55 times more willing than females to try fried insects and meat burgers with larvae on the top [68]. Other studies found that gender was not significantly important [39,72,73,74,75,76,77,78].

In many studies, age was a significant factor, with younger individuals showing more positive attitudes than older individuals towards insect-based food [37,41,50,59,60,62,63,64,66,67,77,78,79,80]. For example, Sogari et al. [78] found that younger individuals were more open to trying insects as food due to their increased awareness of the environmental benefits associated with replacing more conventional animal-based protein with insect-based protein. Their food culture was also less firmly established, suggesting that they would be more willing to try new foods. In contrast, two studies showed that older participants in Japan and China were more likely to eat insects than the younger ones because older people in these countries had previous experience eating insects [81,82], highlighting the importance of long-standing habits. Other studies found that age was not a significant predictor of consumers’ acceptance of insect food [39,52,61,65,68,72,74,76,83].

Individuals with a higher level of education were more inclined to consume insects as food [32,59,62,68,79,84]. Pambo et al. [62] showed that with increasing education levels, Kenyans’ intentions to consume food made from edible insects became firmer in comparison to those of their less educated compatriots. Interestingly, Brunner and Nuttavuthisit [84], in Switzerland and Thailand, found that educational influence differed between cultures, where early adopters of insects as food in Switzerland were more educated, while in Thailand, they were less educated. This was explained by that more highly educated people in Switzerland appeared to care about sustainability and health aspects of entomophagy, while in Thailand, educated individuals instead associated entomophagy with Thailand’s rural traditions. Again, other studies found no link between education and willingness to accept insects as food [39,52,61,67,71,74,83].

Only a few studies found a significant influence on household income and size. Households with higher incomes were more willing to accept insects as food in China [82] and to consume edible insects in Poland [54]. Larger household size in China increased the acceptability of insects as food [82], while in Kenya, larger household size showed decreased acceptability [62]. Interestingly, the rate of acceptance can differ in the same country, as illustrated in three studies. Respondents from the northern regions of Italy showed a higher willingness to accept insects as food than those from the southern regions [65,66], and respondents from Nanjing, China, were more willing to buy edible insects than consumers in Beijing [82].

#### 3.2.2. Psychological Factors

##### Emotions and Attitudes

Emotions can influence attitudes, and both are evaluations of objects. However, while emotions are the evaluation of a state that ceases after the person is no longer in the situation that gave rise to them, attitudes can be temporary or enduring, as they do not necessarily vanish after the person is no longer in that situation [85]. Regarding consumer acceptance of insects as food, these studies showed that the influence of emotions is linked to disgust and curiosity, while that of attitudes is linked more closely to consumers’ concern and interest, food neophobia, and food technology neophobia.

Disgust could generally accrue towards unknown food [86], and in the case of insects as food, it is generally seen as the key negative determinant of consumer acceptance, especially in developed countries. For example, disgust was associated with an unwillingness to accept, try, and pay for both processed and unprocessed edible insects [40,42,51,52,54,59,60,65,75,80,82,83,87,88,89,90,91,92].

There is a difference in the sense of disgust that varied across countries, ranging from 26% to 82%. We have observed that disgust is lower when consumers have been exposed to insect-based products previously. For instance, in Belgium, Van Thielen et al. [93] conducted a study two years after selling insect-based products in the market, where they found that 57% of Belgian participants reported not eating food containing insects. There were many reasons given for this rejection, including price, religion, and diet. However, disgust/feeling dirty were the most important factors, accounting for 26% of these reasons. In a cross-cultural study by Gómez-Luciano et al. [94], only 4% of Dominicans and 25% of Spaniards were willing to accept insect-based protein. For Dominicans, disgust accounted for 82.7% of the reasons given. Notably, this study included other sources of protein (plant-based proteins, mycoproteins, and cultured meat proteins), and insect protein was the least preferred in both countries. 

Curiosity about insects revolved around the taste, texture, and novelty of insects, which was a motivator for consumer acceptance of this food type in their diet. For example, curiosity about insects motivated Swiss and Dutch consumers to try insect-based food [47,95]. For Italian consumers, curiosity also increased their likelihood of future consumption [96], and curiosity relating to taste and texture was a significant factor in encouraging Italian consumers to try cookies made with cricket flour [51]. Moreover, due to the novelty of insects as food, Dutch consumers previously exposed to insects as food were willing to try novel insect products as long as these met their expectations of insect preparation for optimal flavor [97]. 

Interest in insects as food was found by Videbæk and Grunert [60] to motivate Dutch consumers to eat insects in both visible and invisible forms. These consumers were influenced by interest rather than disgust, suggesting that increasing consumers’ interest to encourage the willingness to try insects as food could help to overcome the barriers surrounding entomophagy, for instance, stimulating interest in the health and environmental aspects of eating insects [56,92]. In addition, having variety and novel food experiences were found to increase acceptance of insects as food [31,33,42,56,95,98].

Concerns about the impact of food on one’s health and the environment can influence acceptance. For instance, Italians who are concerned about the health and environmental impacts of insects as food are, on average, approximately 22% more likely to be willing to consume insects than those who are not [64]. Those intending to reduce their conventional meat intake are also more likely to adopt insects as food [67,98].

Food neophobia is a strong predictor of aversion to novel foods, where a high level of food neophobia negatively influences willingness to taste and cook novel food even in young people who are skilled in cookery [99] and vice versa. When food neophobia is low, the willingness to try novel food will be higher [100]. In the case of insect-based food, Modlinska et al. [31] found that people with lower general neophobia and a higher tendency to seek variety tried the insect-labelled samples sooner than people from the other groups. Concerning the studies included in this review, the negative effect of food neophobia on consumer acceptance of insects as food was observed in both developed and developing countries, for example, in Italy [40,63,64,66,75,76,101], Germany [61,73,80,83,92], Poland [54], Switzerland [71], Australia [78], Hungary [98], Taiwan [102], China [82], and Uganda [79]. In addition, cross-country studies concluded similar results [74,84,103,104].

Consumer rejection of food produced using new technology (food technology neophobia) was a significant predictor of consumer acceptance in four studies. Schlup and Brunner [71] found that food technology neophobia negatively affected Swiss consumers’ willingness to accept mealworms, locusts, and caterpillars. It was also likely to decrease Belgians’ readiness to adopt edible insects by 55% [67], their willingness to eat insect burgers and buffalo worms [83], and to discourage Italians from eating insect-based food [64].

##### Social Pressure

Generally, social influence can change an individual’s decisions, as people usually tend to follow others because they like to conform [105]. While few studies have explored the influence of social pressure exerted by peers on consumers’ acceptance of insects as food, there is some evidence from studies analyzing social norms (influence based on others’ evaluations/opinions) and descriptive norms (influence of beliefs about what others do) [106]. Social norms are shown to predict consumer acceptance of edible insects positively or negatively, with low social acceptance and negative social influence from family and friends decreasing consumers’ acceptance and willingness to try insects as food [51,61,96]. Conversely, when peers and experts highly rated the taste of a bar and burger made with mealworms, consumers expected the subjective taste of the products to be of high quality; however, the influence of experts was stronger for those who had low disgust sensitivity toward insect food [49]. Regarding descriptive norms, 53% of students in a tasting session conducted in a study by Jensen and Lieberoth [107] were willing to try roasted mealworms. However, when they thought their colleagues had tried them, the number of students who tried the foods increased to 81%. Interestingly, authors from one study argued that the enjoyment derived from eating with friends can also increase the acceptance of insect-based food when it is associated with fun [108].

##### Wider Availability

The lack of food retailers and specialized shops in which consumers cannot easily find these products is an often-cited barrier to increasing consumption of insects as food. When consumers believed they could easily obtain insect-based products, their determination to purchase or consume them increased [32,62,102]. Conversely, the difficulty in finding these products frustrated intentions to eat insect-based food [65] and was the main reason for not regularly buying insect-based products [95] or eating insects regularly [41]. However, this was not always the case, as observed by Van Thielen et al. (2019) in Belgium two years after the introduction of insect-based food to the market. Only 11% of consumers had tried them, 32% of consumers did not eat them despite their interest in trying them, and 57% did not eat them or show any interest in doing so. This suggests that acceptance may take time, even when products are available in the market, and thus, perceived behavioral control can be an important psychological element to take into account when investigating consumers’ acceptance towards these products.

#### 3.2.3. Familiarity

Familiarity with insects seems to influence consumers’ acceptance positively. Familiarity could arise from entomophagy being rooted in the national culture, such as from an indigenous practice or from food insecurity [109,110]. Association of familiarity with the concept of entomophagy and with food from foreign countries can increase consumer acceptance. When entomophagy is well received from a cultural point of view, as in western regions of Kenya, participants have positive attitudes towards insect-based food [62]. When people become familiar with the concept of entomophagy by learning or hearing about it in a way that builds knowledge about its nature and advantages (such as environmental and nutritional benefits), they are more likely to accept the idea of insects as food [33,44,52,63,67,104]. Not knowing how to prepare and eat insects at home negatively influences acceptance [55,111]. Additionally, familiarity with food from foreign cuisines creates a positive attitude toward eating insects [68], and the lack of previous experience induces a lower willingness to eat insects in China and Germany [74]. This familiarity could differ according to the species of insect that people are accustomed to eating. For example, consumers in northern Uganda accepted the insects proposed in the study (long-horned grasshoppers, flying African termites, and wingless red termites), while in central Uganda, participants only accepted long-horned grasshoppers due to their specific familiarity with the species [79]. In contrast, a lack of familiarity can evoke the idea that eating insects is unnecessary, thereby causing the rejection of insect food. There are various reasons for this, including belief in the sufficiency of meat production [111]; cultural rejection based on the idea that insects are vermin or famine food [33]; a strong food culture whose participants prefer traditional ingredients over novel ingredients, such as Italy [70,112]; or a preference for foods that are familiar to them [71].

### 3.3. Elements of the Marketing Mix

These studies provide interesting commercial insights into the efforts of food technologists and marketers to develop and promote insect-based products. The marketing of edible insects could be facilitated by developing strategies based on an understanding of how marketers can take advantage of the 4Ps of the marketing mix (product, price, promotion, and place) to encourage consumer acceptance of these products [113].

#### 3.3.1. Product

Several studies included in this review have explored aspects of product development, investigating consumers’ acceptance of selected species of insects, sensory attributes, perception of the appropriateness of different food products (carriers), convenience, perception of product benefits, and risk and safety.

The insect species used in the insect-based product can affect the taste, thereby influencing its acceptance [19]. A few studies have explored consumer preferences for insect-based products developed using different insect species; however, the preferred species differed between countries. In Italy, Tuccillo et al. [40] found that crickets, bee larvae, grasshoppers, mealworms, silkworms, and giant water bugs were the most preferred insects. The authors also explored the role of the insect life stage on consumers’ acceptance of insect-based snacks and found that insects in the adult stage were more acceptable than those in the larval stage. In contemporary rural Japan, wasp larvae and grasshoppers were found to be the most acceptable insect types [81]. In Romania, consumers who were willing to eat insects preferred locusts and ants to a variety of products based on other species, including crickets and worms [77]. Finally, the development of insect-based products that are based on species already marketed, such as grasshoppers, mealworms, and crickets, can positively influence consumers’ acceptance [91].

Sensory attributes are crucial in influencing consumer acceptance of new products [16]. Appearance, taste, texture, and color are the most thoroughly explored sensory attributes of the product identified in this review, while flavor was found in three studies only, and results were undetermined. In terms of appearance, several studies have found that consumer acceptance increased when insects were processed and invisible in the final product [30,37,40,41,53,54,61,68,71,77,80,87,111,114,115,116]. Balzan et al. [55] conducted five focus groups to explore the readiness of young Italian to consume insects and found that consumers’ willingness to eat insect-based food decreased when insect parts appeared in the food. However, this may not be the case where entomophagy is common. For instance, in a study conducted in Germany and China, no difference was observed in the willingness of Chinese people to eat both processed and unprocessed insects (e.g., deep-fried crickets, drinks containing silkworm protein, and cookies based on cricket flour), while German participants were more willing to eat processed insect-based food [74].

Taste and texture are critical factors that shape product development and thus consumers’ decisions regarding unfamiliar food [86]. Taste is a significant predictor regardless of whether it is concerned with the conventional meat or insects’ taste. The high importance of the meat’s taste decreases the willingness to adopt insects as an alternative [67]. The taste expectation and experience can determine consumers’ acceptance of edible insects as an alternative source of protein to meat, where individuals’ expectations about the taste of insects can affect their reaction because a good or bad taste expectation can, respectively, increase or decrease the chance that they will eat the insects [68]. Powell et al. [89] found that British consumers’ willingness to pay for insect-based burgers can decrease when they perceive that the taste of insects is bad. Instead, a good taste experience is important for regular consumption [25,95,117]. For instance, in the Netherlands, House [95] revealed that taste was a significant factor influencing Dutch participants’ repeated consumption. In this study, one-third of respondents reported that a good taste would be their reason to buy the product again, one-third indicated that a bad taste experience was the reason for not buying it again, and the remaining participants were ambivalent about the taste.

Texture can also influence individuals’ acceptance of insect-based food positively or negatively [30,47]. For example, the crispy texture of baked insects was preferred by Belgium consumers over the texture of boiled insects [72]. Participants were also more willing to try insect-based food if it was flavored [37]. For example, although Italian participants showed little willingness to accept insect-based food, sweet insect-based products, such as chocolate-coated grasshoppers and cereal bars containing insects, were more attractive than savory alternatives, such as maggot cheese and risotto containing maggots [63]. In Germany, Schäufele et al. [61] concluded that grasshoppers were better liked than mealworms. However, in this study, participants did not try the product. Instead, their preferences were based on a brief description of the way the insects tasted, where grasshoppers were described as neutral-flavored, and mealworms were described as having a flavor similar to nuts. Participants’ decisions may have been shaped by associations between the description and their general taste preferences and not by the actual taste of the insects. Color can also influence acceptance. Bartkowicz and Babicz-Zielińska [53] found that the ground mealworms bar was preferred over the ground crickets bar, which was attributed to the color of the ground crickets bar. Willingness to eat insects as food was found to be influenced by the product’s perceived appropriateness for consumption [118,119]. In the Netherlands, Tan et al. [118] found that acceptance of mealworm products can be influenced by consumers’ perceptions of the appropriateness of the insect-based food product (carriers). The study found that Dutch participants considered meatballs to be appropriate and dairy drinks inappropriate. On that basis, developers of an insect-based product should consider this when choosing food carriers, as different carriers elicited different willingness to pay. According to Lombardi et al. [101], Italians’ willingness to pay differed among three insect-based products in different carriers (pasta, cookies, and chocolate bars), as participants preferred insect-based pasta over insect-based cookies and chocolate bars. This was explained by the likelihood that consumers are more willing to accept insect-based products in savory foods than sweet foods. Food carriers can also influence the intentions of participants in trials. Ardoin and Prinyawiwatkul [44] found that U.S. participants were more willing to try protein bars, chips, snack crackers, or protein shakes, as they perceived these to be the most appropriate of 30 products that included hamburgers, crab cakes, and cheese. Poortvliet et al. [48] showed that consumers were less willing to try a common product made with insects (burger), as it was perceived as less healthy and more disgusting compared to an uncommon product made with insects (skewers). However, we cannot assume that the insect-based burger is an unsuitable product, as it was the third most preferred insect product among 17 insect-based products, following energy shakes and energy bars [93]. In a Belgian study, 37% of participants saw insect-based food as an appetizer, 26% as an addition to the main dish, and 23% saw it as a dessert [72].

The convenience of insect-based food is perceived by consumers as satisfying their desire for a product that is easy to access and cook and fits well with their needs [25,71]. For instance, Verbeke [67] found that consumers were more likely to accept insects as food when these were introduced as snacks because convenience increased their readiness to accept them by 75%.

The perception of the product’s beneficial attributes such as its healthiness, nutritional value, and environmental benefits can influence consumer acceptance. Perceived healthiness and nutritional value can to some extent enhance consumers’ eagerness to try insect-based food [25,46,64,65,71,73,82,93,95,111,115,120,121]. However, perceiving conventional meat as nutritious and healthy can also decrease the willingness to consume insects as food [71]. Perceived environmental benefits of insects as food could increase their acceptance [16,25,46] and enhance subsequent consumption [96]. Regarding the sustainability of insect food production, seven studies found this to be a potential driver of entomophagy [40,46,56,64,65,67,95]. Two other studies, however, found it to be one of the least effective means of motivating acceptance [37,54], while two further studies found it to be an insignificant factor [83,102]. For Cavallo and Materia [114], sustainability was only influential for highly educated consumers. Nevertheless, the increase in the younger generation’s awareness of the unsustainability of food production and consumption may positively influence the perception of the benefits of insect-based products [78].

As far as the novelty of insect-based food is concerned, Clarkson et al. [111] found that the perception of eating insects as new and frightening was the main driver for 16% of participants. Iannuzzi et al. [112] concluded that the novelty of ingredients (pizza made with insect flour) could decrease acceptance, as Italian participants tended to prefer traditional ingredients.

Perceptions of product safety or the potential risk of eating insects could also influence consumer acceptance. Increased perceptions of safety can decrease the sense of disgust [31] and increase the willingness to buy insect-based food [121]. Safety concerns can prevent consumers from eating insects frequently [82], but providing information about the products’ safety can have a positive influence on consumers’ willingness to eat [120] and is important even for regular consumption [41]. If consumers associate eating insects with the risk of contamination and contracting diseases, their acceptance will decrease [25,87], resulting in a lower willingness to pay for insect-based products [101]. Moreover, it could reduce consumers’ willingness to try insect-based foods or eat them frequently [37,82].

#### 3.3.2. Price

Price can shape consumers’ decisions and is often positively associated with consumers’ perception of the product’s quality [122]. However, this element of the marketing mix has mainly been explored in developed countries. In the Netherlands, House [95] interviewed 33 individuals to explore their acceptance of insect-based food, where the price of an insect-based burger (EUR 4) was higher than those of vegetarian (EUR 2–EUR 3) and meat burgers (EUR 1–EUR 3). Although 64% of the participants declared that the price alone would not prevent them from buying the product, it could, in combination with other important factors such as taste, availability, and “fit” with established eating practices, prevent future consumption. Both price and quality were found to have significantly influenced consumers’ acceptance of entomophagy [25]. In an experiment conducted in Germany by Berger [45], 76 participants were exposed to insect-based burgers at two different prices: EUR 2.99 versus EUR 14.99. The higher price had a positive influence on participants’ expectations and willingness to pay for the insect-based burger, as it was associated with the quality of the product and even showed an influence on the later consumption of unprocessed insects (mealworms with truffles) although the price of the truffles was not disclosed. Moreover, price reduction negatively influenced the willingness to pay for the insect-based burger, as it decreased consumers’ expectations of the product’s quality. It was also found that quality alone could influence willingness to try insects such as cockroaches because they were perceived by the participants as poor in quality and spoiled [37].

#### 3.3.3. Promotion

Adapting the design and promotion of insect-based food to consumers’ needs, emotions, and attitudes is crucial to increasing their acceptability. Promotional communication can be developed on the perception of the product’s benefits, safety, and risks. Communicating with consumers about the benefits of eating insect-based food (e.g., chocolate bars made with protein from crickets), whether delivered as social or individual benefits, increases their willingness to eat these products [70]. Moreover, advertising insects as healthy and sustainable can gain favorable attention from consumers [93]. In New Zealand, Clarkson et al. [111] ran focus groups with 32 participants, aiming at designing the ideal insect-based product with attributes that consumers would most strongly prefer. The participants designed it to be promoted as a convenience food (e.g., in snacks), emphasizing the idea that insects are healthy and recommending that it should be sold at a premium price and in sustainable packaging that would support the idea of insect sustainability. Van Thielen et al. [93] concluded that it should be declared on the packaging that the product contained insects, as this could increase the willingness to pay for the insect-based product [115]. It has been suggested that insect-based products should be promoted by affective messages such as an invocation of the positive emotions that arise from choosing insect-based food, as it is good for one’s health, instead of cognitive messages stating that research shows that insect-based products are healthy and environmentally friendly [123]. However, three studies found that nutritional claims, for example, that it was high in protein, decreased consumers’ acceptance of insect-based food [78,114] Communication of information about the safety or potential dangers of the product may or may not lead to its rejection [86]. Therefore, when insect-based food is promoted, it should be clearly established that these products are safe to eat [120,124], as this can positively influence consumers’ willingness to eat them, which is important for regular consumption [41].

#### 3.3.4. Place

In terms of locations for buying these products, the availability of insect-based products is one of the important factors that can determine consumer acceptance [125]. Participants in different studies appear to prefer supermarkets, followed by health food stores, restaurants, and kiosks [93,111,120].

## 4. Discussion

### 4.1. General Discussion

The current systematic review offers a comprehensive overview of consumer acceptance of insects as food, as it includes research on developed and developing countries, covers both quantitative and qualitative studies, and used the SPIDER tool when developing the inclusion/exclusion criteria. Our study contributes to previous studies [24,25,26] by developing a framework that helps to discuss more in detail the impact of psychographics and marketing aspects on consumer acceptance of insects as food, thus providing insights to marketers and other stakeholders of the food industry.

Our results confirm most of the results of previous systematic reviews even if we observe that they varied in the number of factors identified and in the level of significance of two important factors: age and education. According to ref. [22,23], younger and more educated people were more willing to accept insects as food, while another systematic review [21] concluded that age and level of education were non-significant in the majority of studies. Our findings support that younger and more educated people are more likely to accept insects as food, but we also observe that, in several surveys, the distribution of participants’ age was biased towards young people. We also observe that although males were found to be more willing than females to accept insects as food, this difference varied between countries [23], and many studies showed it was not significant [21]. We concur with findings that question the influence of these socio demographic characteristics on consumer acceptance and suggest that future research further explore their role, especially in developing countries where only a few studies have investigated this aspect.

The inclusion of developing countries has revealed that consumers from these nations are more willing to accept insect-based food than people in developed countries. However, we observed the opposite in two cases, which were due to the influence of familiarity and religion. Familiarity plays a significant role in Japan: although it is a developed country, insect-based products were more acceptable to the older citizens, who were familiar with eating insects. This suggests that developed countries can learn from developing countries where people are more familiar with the consumption of insects as food, and thus, more studies could be conducted in these areas of the world. Multiple cross-sectional studies involving developed and developing countries can help to understand both cross-cultural differences and how familiarity can increase consumers’ acceptance of this food. Religion is another important factor: for example, although India is a developing country, the acceptance rate among Indians was lower than that of participants from the USA. This was attributed to the perception that insects are prohibited to eat in India from a religious perspective, where 74% are Hindu, 10% Catholic, 10% Muslim, and 6% other, while 16% of the American participants had previous experience of eating insects [90].

Regarding methods of marketing insect-based products, first, it is important when designing a product to bear in mind attributes that will increase the likelihood of acceptance. Second, marketing strategies must be developed to communicate benefits of insects as food to specific group of consumers. Consequently, the rest of the discussion is centered on the two previous dimensions.

### 4.2. Insights to Develop Insect-Based Products

Our results show that consumers are attracted by products that contain processed insects in a convenient form. Developing products that consumers find familiar in terms of food carriers and taste (e.g., cake, muffin, and pasta) can enhance their acceptance because they decrease the sense of novelty of the product, which in turn may reduce food neophobia [31,125].

The marketing of these products should take advantage of health and environmental benefits, which could be communicated with voluntary labels and using sustainable packaging. In addition, it should be clear on the packaging that the product contains insects. We could not draw any conclusion regarding preferred sensory attributes, insect species, and appropriateness of food carriers because of the consumers’ heterogeneity of preferences as observed in different cultures. This means that insect-based products should be developed in accordance with consumers’ preferences in their respective countries [126].

Price is a crucial factor that can influence consumer purchasing decisions. High prices usually lead to lower the demand of these products, especially in poor countries that suffer from food insecurity [127]. The marketing of insects as food with high prices could also reduce repeat consumption, but studies included in this review suggest that high prices will not prevent consumer acceptance. This could be attributed to the fact that the majority of studies included were conducted in developed countries and that some consumers associate high prices with high quality [122]. Although only a few studies have explored the role of the purchasing place, the promotion of insect-based food on a large scale could be facilitated by multiple retailers, where consumers can easily access the product.

### 4.3. Marketing Strategies

Based on the evidence presented in our review, we suggest four strategies when promoting insect-based food. First, these products should be initially marketed as processed safe, healthy, and environmentally friendly food and promoted by public campaigns, scientists, and experts because trust towards these sources of information can enhance consumer acceptance. Thus, the Western food industry and retailers should invest more in research and development to produce processed insect food, which is familiar to food items that are more palatable than unprocessed insects. This could be a winning product development marketing strategy that creates new products that firms can target at their existing markets. Instead, food companies in developing countries could opt for a market development strategy promoting their processed insect food products into new foreign markets using existing offerings with minimal product development. Despite risks that companies might face for pushing these marketing strategies, lessons of diversification from the past show that protein initially seen as unconventional may become popular as, for example, sushi in the West and the American experience of lobster [41].

Second, when marketing insect-based products, it is important to distinguish between different segments of consumers [16]. One key result of the present review is that early adopters of insect-based food are a specific segment of consumers consisting of young males and well-educated people. Marketing strategies targeted at early adopters could help marketers to generate social pressure on other groups of consumers who might accept the consumption of these products. Our findings are in line with those of Mancini, Moruzzo et al. [22], and Onwezen et al. [23] and with the characteristics of early adopters of Rogers’ innovation-adoption curve (2003), as they include young educated people. In addition, well-educated people show more concern about the environment [114], and thus, they are more open to the consumption of insect-based foods as opposed to conventional meat-based foods. Regarding gender, while males are more willing to accept novel food, females seem to have a stronger aversion than males to insect-based foods, as they might be more concerned about the safety of novel food [128]. However, in other studies, the influence of gender is indetermined. For example, during the COVID-19 pandemic, Khalil et al. [124] found that consumers in favor of eating insects before and during the pandemic were young, highly educated people who were employed with a low level of income, but no difference between males and females was observed in terms of willingness to consume insect-based products. Thus, age and education appear to be more stable factors in affecting consumer acceptance, as they did not change their influence even during the pandemic. Our findings also indicate that individuals who are familiar with entomophagy are early adopters, and those who care about making food choices that are healthy and environmentally friendly are curious people who seek novel experiences. Our results are in line with Olabi et al. [100], who found that those who were exposed to other ethnic foods and had travelled outside their home countries have less food neophobia than those who did not have these experiences. That being said, the opposite was observed by Onwezen et al. [123], who concluded that people with weak personal norms on health and the environment are more likely to try insect-based food when adopting affective messages.

Third, encouraging unwilling consumers by creating a positive experience can increase their willingness to try these products, can increase future consumption, and can even mitigate the feeling of disgust, which is one of the major barriers to entomophagy. This is a marketing strategy that the food industry and policy makers could push with children, as their food preferences may be more pliable than other segments of consumers. Targeting children with food school programs could be a good way to change the attitudes of new generations towards the consumption of insects, but our systematic review has shown that while many studies have interviewed higher education students, there is a lack of studies where scholars have investigated consumer acceptance of children [41,129,130].

Fourth, educating consumers by providing them with information about insect-based food can enhance their willingness to try it [109]. Information campaigns about the benefits of entomophagy can increase not only consumers’ willingness to try insects [39] but even their willingness to pay a premium price [101]. More research is needed on the role of informative sessions, as the conflicting results in the existing studies may depend on the kind of information with which participants were provided. Creating familiarity in unwilling consumers can increase their acceptance by creating awareness of insects as food, which can be created by exposing consumers to insects as food by educating them about the potential benefits of insects in addition to giving them the chance to try it [16], [131]. Familiarity can be created by exposing people to insects as food in a way that can build memories though whether or not this will lead to acceptance of the food will depend on the nature of the remembered experience, which may be good or bad [97]. A positive experience of trying insects can increase willingness to consume them later [64,66,69,71,83,132]. For instance, 85% of Australian consumers who had tried insects as food before were willing to try them again [25].

Fifth, using nudge strategies that would “alert people’s behavior in a predictable way without forbidding any option or significantly changing their economic incentives” [105], which is especially useful when it comes to making decisions in unfamiliar situations. This strategy was found to be effective in changing students’ food choices when it came to healthy food such as whole wheat bread [133]. This was found to be the case in one study by [134], where they concluded that the willingness to try edible insects can be increased by using a combination of social norms (providing information about social responsibility regarding environmental protection and sustainability) and environmental boost (providing information regarding the benefits and the positive aspects of entomophagy such as food security). Therefore, we suggest nudging consumers to accept insects as food by exposing them to the product via tasting or informative sessions. However, using nudging is just one way to motivate people and encourage them to make better decisions from the policymaker’s perspective. In addition, we should not be overoptimistic about the role of nudges because DellaVigna and Linos [135] conducted a meta-analysis of 26 studies published in academic journals, and they questioned the effectiveness of using nudges because of reasons such as the chance that these studies used different kinds of trials and publication bias, as it easier to publish significant statistical findings.

Finally, a SWOT (Strengths, Weaknesses, Opportunities, and Threats) analysis can provide insights to decision makers to better plan marketing strategies that can shape the development of an insect-based food market. This analysis could be applied to the development of both traditional and novel foods [136]. For the internal factors of the SWOT analysis, our results and other studies indicate that food processors who wish to develop insect-based food can take advantage of the strengths of these products, i.e., a healthy alternative to meat rich in protein, environmentally friendly, and needing less land and water than conventional meat production. However, insect-based food is a novel product, and this can be considered a weakness for producers as the development of new supply is a risky, long, and challenging process, especially in the light of a low consumer acceptance. Concerning external factors, developers need to take the opportunity to market these products by targeting early adopters with processed products resembling well-known food carriers. However, despite the fact that the development of these products is a risky activity because other alternative sources of protein (plant-based food and cultured meat) seem to be more preferred by consumer than insect-based food, the threat of not investing in these products can be dangerous both for the food industry in terms of competitiveness and for our planet in relation to sustainability. Therefore, it is necessary to conduct more interdisciplinary research to obtain a better understanding of consumer acceptance of insect-based food and expend more effort on behalf of policy makers to develop standards and regulations that can facilitate the commercialization of these products.

### 4.4. Limitations

Our study has three limitations. First, although we aimed to provide a comprehensive analysis in this review, our findings can mostly be applied to consumers from developed countries. In addition, we included only English-language publications, and consequently, our results are limited in scope. Second, the articles included in this review focused more on socio-demographic and psychological factors in relation to product attributes and promotion. Future research should focus on the other factors that can influence acceptance (i.e., price and place), thereby improving the developed framework so that it can be more widely applied. Third, the focus of this review was on consumer acceptance, but other challenges should be taken into consideration, such as safety and production costs, as insect-based food is a novel product. Therefore, it will be important to conduct studies that tackle more than one aspect to give a comprehensive view that will be more helpful to stakeholders.

Despite these limitations, our findings have implications that are useful for policymakers, producers, and retailers who seek to encourage consumers to change their choices. Policymakers have a responsibility to mitigate such a major issue by considering that people tend to trust authorities more than companies. For policymakers, particularly in Islamic countries, it is important to investigate the acceptability of insects as food from a religious point of view, as some insects (e.g., crickets and mealworms) are not religiously acceptable according to various schools of Islamic thought, such as Al-Shafi’i and Al-Hanbali [137]. Producers and retailers can benefit from the outcomes of this study by designing and developing marketing strategies for insect-based products, taking into consideration the potential concerns and heterogeneity of preferences that appear to be associated with consumer acceptance.

## 5. Conclusions

Edible insects could contribute to solving major global issues such as food insecurity and global warming. However, consumer acceptance remains low at least in Western and developed countries, where most reviewed studies were conducted. As a result of this review, we were able to develop a framework that highlights factors increasing consumer acceptance of insects as food. The findings also allowed us to consider marketing strategies that can be developed to promote the growth of these new food markets. However, the implementation of these strategies is challenging because they must be tailored to specific groups of consumers, they vary from country to country, and are risky from an economic point of view. More research is needed to explore the potential market for this alternative source of protein in different contexts, both in developed and especially in developing countries, which are more likely than rich countries to be severely affected by food insecurity and climate change.

## Figures and Tables

**Table 1:** The inclusion and exclusion criteria for article selection using the SPIDER tool.

Criteria	Inclusion	Exclusion
S: Sample	Consumer studies	Publications not in English
PI: Phenomenon of Interest	Focus on consumer acceptance of insects as food	Focus on consumer acceptance of other alternatives (e.g., artificial meat and plant-based)
D: Design	Choice experiment/survey/interview/focus group/questionnaire/case study	-
E: Evaluation	Acceptance/preferences/perception/values/attitudes/reaction/behavior/consumption/liking/willingness to accept/willingness to purchase/willingness to pay/willingness to buy/willingness to try	-
R: Research type	Qualitative/quantitative/mixed method	-
Other criteria		Reviews/books

## Data Availability

Information about data presented in this study is available in the supplementary material.

## Supplementary Materials

The following supporting information can be downloaded at: https://www.mdpi.com/article/10.3390/foods12040886/s1, Table S1: The characteristics of the samples and the main factors of the reviewed articles; Table S2: Details of the products reviewed.
