# Supplementary material for: Insects as Food: Consumers’ Acceptance and Marketing
Source: Foods. 2023 Feb 19;12(4):886. doi: 10.3390/foods12040886 (PMC9956212; doi:10.3390/foods12040886)
Supplement: Supplementary file 1 [file foods-12-00886-s001.zip › foods-2157610-supplementary.pdf]

Table S1: The characteristics of the samples and the main factors of the reviewed articles

Authors	Country	Sample size: female; mean age (age range)	The technique (year)	Main factors
1. Alemu et al., (2017)	Kenya	611: 51%; 40.02 (18-85)	Choice experiment (Dec 2014-Jan2015)	Preferences for nutritional value and food safety information, recommendations by officials, shopping places
2. Ardoin & Prinyawiwatkul, (2020)	US	1005: 68.4%; ns (over 18)	Online survey (ns)	Product appropriateness, unfamiliarity with insects as food
3. Balzan et al., (2016)	Italy	32: 65.6%; 24.5 (20-35)	5 focus groups (ns)	The form in which the products are presented, lack of practice in preparation
4. Barsics et al., (2017)	Belgium	135: 23%; 19.4 (17-25)	Experiment (ns)	The information session about entomophagy (encompassing ecological, health, and gastronomic aspects of entomophagy)
5. Bartkowicz & Babicz-Zielińska, (2020)	Poland	101: 73%; ns (ns)	Experiment (ns)	Visibility of insects, the colour of the ground crickets in the bars
6. Barton et al., (2020)	Canada	Survey 107: 57%; ns (19-69)<br>Experiment 102: 58.8%; ns (19-69)	Survey<br>Experiment (ns)	Tasting session
7. Berger et al., (2018)	Germany	Total 240<br>**S1** 80: 40%; 22.61 (ns)<br>**S2** 160: 40%; 23.3 (ns)	Experiments (ns)	Price and expected quality
8. Berger et al., (2019)	Switzerland and Germany	**S1** 120: 50%; ns (ns)<br>**S2** 90: 45%; 21.39 (ns)	Experiments (ns)	Peer and expert rating of insect food
9. Brunner & Nuttavuthisit, (2019)	Switzerland and Thailand	Total 1042<br>Switzerland 542: 56%; 54 (ns)<br>Thailand 500: 54%; 44 (ns)	Questionnaire (ns)	The level of education and food neophobia
10. Caparros Megido et al., (2014)	Belgium	189: 44.4%; ns (<13 ≥ 45)	Experiment (ns)	The texture, types of meal

11. Caparros Megido et al., (2016)	Belgium	79: 56%; ns (18-25)	Experiment (2014)	Gender, previous knowledge of entomophagy and previous experience
12. Castro & Chambers, (2019)	USA, England, Mexico, India, Japan, China, Russia, Spain, South Africa, Australia, Brazil, Peru, Thailand	Total 7560: ns; ns (18 to more than 55)	Online survey (ns)	The appearance of insects' body parts, the idea is disgusting
13. Cavallo & Materia, (2018)	Italy	135: 46%; ns (18-35)	Experiment (ns)	Visibility of the insect shape, high-protein claim
14. Chang et al., (2019)	Taiwan	316: 41.1%; ns (31-50)	Survey (ns)	Consumers' attitudes, perceived behavioral control, food neophobia
15. Cicatiello et al., (2016)	Italy	201: 55%; 43 (14-78)	Survey (2015)	Familiarity with food from a foreign cousin, gender, education
16. Cicatiello et al., (2020)	Italy	62:47%: 24 (18-35)	Experiment (ns)	The importance of the taste of food, familiarity with foreign food, gender, education
17. Circus & Robison, (2019)	UK	Interviews 7: (sociodemographic not collected)<br>Survey: number not reported (sociodemographic not collected)	Interviews and an online survey (ns)	Disgust, environmental friendliness
18. Clarkson et al., (2018)	New Zealand	32: 71.8%; ns (18-75)	Focus groups (ns)	Culture, lack of need for an alternative to meat, and lack of knowledge on how to prepare and eat them at home
19. Collins et al., (2019)	UK	161 children: 35%; ns (6-15)<br>114 children's parents: 58%; 45 (33-75)<br>1020: 65%; 21 (12-90)	Group activity (ns)<br>Questionnaire(ns)<br>Choice experiment (2015)	Visibility of insects

20. de Koning et al., (2020)	China, US, France UK, New Zealand, The Netherlands, Brazil, Spain, The Dominican Republic	Total: 3091: 59.3%; 34.1 (16-83)<br>China 571: 60.8%; 31.2 (19-72)<br>US 539: 75.4%; 44.1 (18-71)<br>France 484: 31.8%; 29 (18-68)<br>UK 366: 76.2%; 32 (19-67)<br>New Zealand 268: 53.2; 37.9 (18-70)<br>The Netherlands 231: 62.3%; 29.6 (17-70)<br>Brazil 216: 56.9%; 38.2 (17-77)<br>Spain 210: 48.1%; 35.1 (19-83)<br>The Dominican Republic 206: 66%; 26.2 (16-96)	Survey digital and hard copy (2018-2019)	Food neophobia
21. Dupont & Fiebelkorn, (2020)	Germany	718: 57.5%; 13.67 (9-19)	Questionnaire in a paper-pencil format (2018)	Age, food neophobia, attitudes
22. Fischer & Steenbekkers, (2018)	The Netherlands	140: 54%; 24.9 (ns)	Online survey (2014)	The most marketed insects, affective attitude component and disgust
23. Gere et al., (2017)	Hungary	400: 65%; 25.5 (ns)	Online survey (2016)	Food neophobia, seek new food choice options, intention to reduce their intake of fresh meat in the coming year
24. Gómez-Luciano, Vriesekoop, et al., (2019)	Dominican Republic and Spain	Total 401<br>Dominican Republic 201: 31.5%; 25 (16-70)<br>Spain 200: 47%; 35.5 (16-83)	Online and face to face survey (2017)	Disgust
25. Gómez-Luciano, de Aguiar, et al., (2019)	UK, Spain, Brazil Dominican Republic	Total 729:<br>UK 180: 51.7 %; ns (>24 ≥ 65)<br>Spain 200: 47%; ns (>24 ≥ 65)<br>Brazil 216: 56.9%; ns (>24 ≥ 65)<br>The Dominican Republic 133: 50.4%; ns (>24 ≥ 65)	Survey (2017)	Disgust

26. Hartmann & Siegrist, (2016)	Switzerland	Total 104<br>Control group 53: 41.5%; 32.3 (18-65)<br>Experimental group 51: 45.1%; 35.4 (18-65)	Experiment (2015)	Exposure to processed insect products
27. Hartmann et al., (2015)	Germany and China	Germany 502: 52%; 44.3 (20-69)<br>China 443: 51%; 44.2 (20-69)	Survey (2014)	Food neophobia, taste expectations, social acceptance, and past experience with eating insects
28. House, (2016)	The Netherlands	33: ns; ns (ns)	semi-structured interviews (2015)	Taste, availability, degree of fit with current eating patterns
29. Iannuzzi et al., (2019)	Italy	587: ns; ns (18-56)	Online survey (ns)	Disclosed product ingredients
30. Jensen & Lieberoth, (2019)	Denmark	189: 84%; 21.7 (ns)	Online survey, sensory test (ns)	Perceived social norms
31. Kornher et al., (2019)	Germany	311: 73.2%; 30.85 (ns)	Choice experiment (2016)	Disgust, Food neophobia. interest in consuming climate-friendly products
32. La Barbera et al., (2018)	Italy	118: 49%; 23.95 (ns)	Experiment (ns)	Food Neophobia and disgust
33. La Barbera et al., (2020)	Denmark and Italy	**S1** Denmark 975: 51%; ns (18-75)	Focus groups (32), then online questionnaire (2017)	Disgust, individuals' interest to try novel experiences and eating novel foods
		**S2** Italy: 543: 60%; 28 (ns)	Online survey (ns)	
34. Lammers et al., (2019)	Germany	516: 51.6%; 47 (18-87)	Online survey (2018)	Disgust, previous insect consumption and food neophobia
35. Laureati et al., (2016)	Italy	Survey 314: 65.4%; 31.9 (18-80)<br>Experiment (68 of the above): 61.8%; 21.4 (ns)	Online survey, experiment (ns)	Food neophobia, age, gender, cultural background

36. Le Goff & Delarue, (2017)	France	100: 67%; ns (18-64)	Nonverbal evaluation (videotape before and after taste evaluation) (ns)	Tasting
37. Lensvelt & Steenbekkers, (2014)	The Netherlands and Australia	**S1** 209: 134 Netherlands & 75 Australia; ns; ns (ns)<br>**S2** 133: Australians 63.3%; ns (<10 > 80)	Online survey Experiment (ns)	Previous experience, price, quality, perceived product benefits and risk, convenience, trust
38. Liu, Ai-Jun; Li, Jie; Gómez, (2020)	China	614: ns; ns (18 - more than 65)	Survey (2012)	Disgust, insect phobia, safety concerns, age, income, region, household size
39. Lombardi et al., (2019)	Italy	200: 40%; 20.5; (ns)	Experiment (2017)	Different carriers, disclose information concerning the benefits, food neophobia, beliefs and attitudes about insects
40. Mancini, Sogari, et al., (2019)	Italy	165: 83.3%; ns (ns)	Experiment (2018)	Food neophobia, the intention to eat products containing insect powder in the coming months
41. Menozzi et al., (2017)	Italy	231: 61.9%; 23.6 (young adults)	Online survey (ns)	Attitude, perceived behavioral control, beliefs regarding health and the environment, disgust arising from seeing insects around
42. Modlinska et al., (2020)	Poland	99: 81.8%; 22 (18-45)	Experiment: Trying food containing insects and semi-structured individual interview (2019)	Labelling, general neophobia, and variety-seeking tendency in food consumption
43. Motoki et al., (2020)	Japan	**S1** 96: 32.3%; 41.1 (ns)<br>**S2** 104: 30.7%; 42.9 (ns)<br>**S3** 104: 49%; 39.9 (ns)	Online survey (2020)	Social companions (friends), location (pubs and food festivals)

44. Myers & Pettigrew, (2018)	Western Australia	77: 87%; 73 (60-100)	Interviews (April 2015-February 2016)	Perceived cultural norms, lack of necessity for eating insect food, and concerns about the natural balance
45. Nyberg et al., (2020)	Sweden	Questionnaire 82: 64.6%; ns (more than18)<br>Workshop 15: 40%; ns (ns)	Questionnaire (2018)<br>Workshop discussion (2019)	Concerns about the environment and health, willingness to try something “exciting"
46. Olum et al., (2020)	Uganda	310: ns; ns (ns)	Face-to-face interview using structured questionnaire (ns)	Culture, familiarity with edible insects, age, education, food neophobia
47. Onwezen et al., (2019)	The Netherlands	**S1** 2461: 41.1%; 46 (ns)<br>**S2** 2771: 50.2%; 45.9 (ns)<br>**S3** 1001: 48.9%; 49.6 (ns)	Experiment (ns)	Weak personal norms regarding personal health and being environmental-friendly, affective communication
48. Orkusz et al., (2020)	Poland	Total: 866<br>Survey 464: 64.8%; ns (18-24)<br>Sensory test 402: ns; ns (18-78)	Survey<br>Sensory test (2019)	Food neophobia
49. Orsi et al., (2019)	Germany	393: 51%; 36 (13-82)	Online survey (Dec 2018-Jan 2019)	Visibility of the insects. Food neophobia and disgust
50. Palmieri et al., (2019)	Italy	456: 67.9%; 41 (18-65)	Web-based survey (2018)	Taste expectations, concerns about the health and environmental impact of insect food, previous experiences with edible insects, neophilia, food technology neophobia
51. Pambo et al., (2017)	Kenya	54: 53.7%; 45 (ns)	Laddering interviews (ns)	Providing information, tasting cricket buns
52. Pambo, Okello, et al., (2018)	Kenya	432: 55.6%; 28.1 (ns)	Survey (ns)	Trust in government and industry, perceived availability of insect-based foods, household size, level of formal education

53. Pascucci & De-Magistris, (2013)	The Netherlands	122: 51% (18 - over 64)	Choice Experiment (2011/2012)	Visibility, logo showing insects, nutritional claims, information about the health and environmental benefits
54. Payne, (2015)	Japan	Survey 220: 42%; ns (ns)<br>Interviews number not reported: ns; ns (ns)	Sequential mixed methods approach (2013)	Age, availability of the species
55. Petersen et al., (2020)	US	Survey 98: 51%; 20 (18-24)<br>Tasting test 61: ns; ns (ns)	Experiment (ns)	Environmental and nutritional benefits associated with insect food products
56. Piha et al., (2018)	(Northern and Central Europe- Finland, Sweden, Germany, and the Czech Republic)	Total 887:<br>Northern Europe 430: 60%; 37.5 (17-96)<br>Central Europe 457: 61%; 39.7 (17-96)	Online survey (2016)	Consumer knowledge (subjective and objective). Product-related experiences, food neophobia. general attitudes
57. Poortvliet et al., (2019)	The Netherlands	130: 68%; ns; (18-65)	Experiment (ns)	Use of insects in common product type
58. Powell et al., (2019)	UK	510: 50%; 34.33 (18-70)	Experiment (ns)	Disgust propensity, perceive taste and naturalness
59. Roma et al., (2020)	Italy	310: 61.1%; 33 (18-81)	Online survey (2019)	Age
60. Ruby & Rozin, (2019)	US<br>India	275: 55%; 35.9 (ns)<br>201: 34%; 32 (ns)	Questionnaire (ns)	Disgust, Religion, sushi consumption, benefits
61. Rumpold & Langen, (2019)	Germany	149: 55%; 31.9 (10-69)	Survey<br>Sensory test (2017)	Providing participants with information about edible insects
62. Schösler et al., (2012)	The Netherlands	1083: 50%; 49.5 (18- 92)	Online survey (2010)	Visibility of insects
63. Schäufele et al., (2019)	Germany	342: ns; ns (under 18-over 65)	Survey (ns)	Species, low social and cultural acceptance, visibility of insects
64. Schlup & Brunner, (2018)	Switzerland	379: 54%; 53 (ns)	survey (2015)	Convenience orientation, discernibility of insects in food, perceived health benefits of meat, food technology neophobia, prior consumption, need for

				familiarity, food neophobia, expected food healthiness of insects, gender
65. Séré et al., (2018)	Burkina Faso	360: 48%; ns (15 and 65)	Semi-structured interviews (2015-2016)	Availability of the species and its host, ethnic groups
66. Simion et al., (2019)	Romania	122: 44%; ns (over 20)	Online survey (ns)	Visibility
67. Sogari et al., (2017)	Italy	109: 53%; ns (18-25)	Experiment (2015)	Curiosity about the taste and texture, disgust at insects, social influence by family members and/or friends
68. Sogari et al., (2018)	Italy	88: 51%; ns (ns)	Experiment (ns)	Tasting the insect-based products
69. Sogari et al., (2019)	Italy	88: 51%; 25.7 (18-40)	Experiment (2016)	Gender, food neophobia, intention to try
70. Sogari, (2015)	Italy	46: ns; ns (ns)	Experiment (2015)	Curiosity, the environmental benefits, family members' and friends' opinions
71. Sogari, et al., (2019)	Australia	555: 49.7%; ns (18-40)	Open-ended questions (2018 and 2019)	Neophobia and disgust, perception of threats to masculinity
72. Szendrő et al., (2020)	Hungary	414: 65.5%; ns (18->50)	Online survey (2020)	Gender, disgust regarding food made from insects, education
73. Tan et al., (2015)	The Netherlands and Thailand	Total 54: 64.8%; 38 (20-65)<br>Dutch 29: 62%; ns (ns)<br>Thailand: 25; 59%; ns (ns)	8 Focus groups (ns)	Cultural exposure, individual eating experience
74. Tan et al., (2016)	The Netherlands	103: 39.8%;22.9 (ns)	Experiment (ns)	Perceived food appropriateness
75. Tan, Tibboel, et al., (2017)	The Netherlands	100: 34%; 23.3 (ns)	Experiment (ns)	Perceived food appropriateness
76. Tan, Verbaan, et al., (2017)	The Netherlands	Total 214<br>135 willing tasters: 80%;33 (18-65)	Experiment (ns)	Perceive food preparation appropriateness, satisfied with the taste experience

		79 unwilling tasters: 65.8%; 50.9 (18-65)		
77. Tuccillo et al., (2020)	Italy	The survey 400: 53.7%; 39 (18-75)<br>The sensory evaluation 58: 36%; 38.3 (19-67)	Survey<br>Sensory evaluation (2020)	Gender, food neophobia, disgust, visibility
78. Van Thielen et al., (2019)	Belgium	388: 50%;43.5;(18-69)	Telephone survey (2016)	Packaging, place, promotion
79. Vanhonacker et al., (2013)	Belgium	221: 64.3%; 41.3 (18->60)	Online survey (2011)	NA (there were other substitutes in the survey and all respondents were negative toward insects)
80. Verbeke, (2015)	Belgium	368: 61%; 42 (18-79)	Online survey (2013)	Food neophobia, convenience orientation, the importance of the environmental impact of food choices
81. Verneau et al., (2016)	Denmark and Italy	Total 264<br>Denmark 136: 44.8%; 23.33 (ns)<br>Italy 128: 55.4%; 23.94 (ns)	Experiment (ns)	Communicating with consumers with different messages (about the benefits for society and individuals)
82. Verneau et al., (2020)	Italy and Denmark	280: 49%; 23.61 (ns)	computer-based questionnaires (ns)	Perceived behavioral control, gender, education
83. Videbæk & Grunert, (2020)	Denmark	975: 50.9%: ns (18-75)	Choice experiment (2017)	Interest in edible insects as food. Disgust, age, gender
84. Wilkinson et al., (2018)	Australia	820: 45%; ns (18-65)	Online survey (ns)	Taste/flavor, the appearance of insects, safety, quality
85. Woolf et al., (2019)	USA	397: 65.7%; ns;(18-94)	Online survey (2017)	Familiar with the concept (heard, seen, learned), knowledgeable about the benefits

ns: not specified.

Table S2: Details of the products reviewed

**Authors**	**Country**	**Products Details**	**Visible/invisible**	**Insect type**
1. Alemu et al., (2017)	Kenya	Termite powder, whole termites fried and salted. Both were presented with Ugali (stiff porridge)	Visible and invisible	Termites
2. Ardoin & Prinyawiwatkul, (2020)	US	A list of 30 products includes protein, energy bars, chips, snack crackers, protein shakes, bakery, cereal products, snacks, candy	Visible and invisible	NS
3. Balzan et al., (2016)	Italy	Cheddar cheese larvets, lollipops, chocolate-covered scorpion, worm salt, dried crickets, baked grasshoppers, toasted scorpions	Visible and invisible	Larvae, Scorpions, worms, crickets, grasshoppers
4. Barsics et al., (2017)	Belgium	Bread faux-labelled as containing 10% mealworm flour	Invisible	Mealworms
5. Bartkowicz & Babicz-Zielińska, (2020)	Poland	insect bar with whole mealworms, another with ground mealworms, and a bar with crushed crickets	Visible and invisible	Mealworms, house crickets
6. Barton et al., (2020)	Canada	Drink contains cricket-based protein powder	Invisible	Crickets
7. Berger et al., (2018)	Germany	Mealworm burger and mealworms with truffles	Visible and invisible	Mealworms
8. Berger et al., (2019)	S1 Switzerland<br>S2 Germany	**S1** mealworm nutrition bar<br>**S2** mealworm nutrition bar and mealworm burgers	Invisible	Mealworms
9. Brunner & Nuttavuthisit, (2019)	Switzerland<br>Thailand	Insect burger, crunchy larvae and chips made with cricket flour, muesli with insects for breakfast and sweet insect mousse as a dessert	Visible and invisible.	Larvae, crickets
10. Caparros Megido et al., (2014)	Belgium	Edible insects (baked/boiled/flavoured or dunked in chocolate)	NS	Mealworms, house crickets
11. Caparros Megido et al., (2016)	Belgium	4 burgers: beef, lentils, mealworms and beef, mealworms and lentils	Invisible	Mealworms
12. Castro & Chambers, (2019)	13 countries: USA, England, Mexico, India, Japan, China, Russia, Spain, South Africa,	Foods containing insect powder as an ingredient	Invisible	NS

	Australia, Brazil, Peru, Thailand			
13. Cavallo & Materia, (2018)	Italy	Snacks with the shape of an insect, and snacks made with insect flour	Visible and invisible	NS
14. Chang et al., (2019)	Taiwan	Cricket biscuits, cricket bread, fried insects (e.g., grasshoppers, pupae, mealworms)	Visible and invisible	Crickets, grasshoppers, pupae, mealworms
15. Cicatiello et al., (2016)	Italy	Preparation comparable to sushi, street food stands with different types of fried insects, skewers with pupae, plate with larvae and pupae with some vegetables, meat burger with some larvae on the top	Visible.	Pupae, larvae
16. Cicatiello et al., (2020)	Italy	Chocolate bar with insect flour, whole crickets, tortilla chips containing insect flour, and dried whole mealworms with caramel	Visible and invisible.	Crickets, mealworms
17. Circus & Robison, (2019)	UK	Edible insects	NS	NS
18. Clarkson et al., (2018)	New Zealand	Variety of products developed by participants (i.e., sweet snack, drink, or breakfast options)	Visible and invisible	Locusts, crickets
19. Collins et al., (2019)	UK	Variety of insect-based products: e.g., Insect bar, cookies with cricket powder, bug salad, fried rice with larvae and Insect quiche, mealworm protein with rice, insect burger, mealworm mince, grasshopper mince	Visible and invisible	Mealworms, locusts, crickets, larvae, bugs, grasshoppers
20. de Koning et al., (2020)	China, US, France UK, New Zealand, The Netherlands, Brazil, Spain, The Dominican Republic	Insect-based protein	NS	NS
21. Dupont & Fiebelkorn, (2020)	Germany	Insects as food, insect-based burger	NS	NS

22. Fischer & Steenbekkers, (2018)	The Netherlands	17 species of insects	NS	Grasshoppers, mealworms, butterflies, dragonflies, caterpillars, crickets, beetles, moths, bees, termites, worms, water bugs, cockroaches, ants, wasps, insect eggs, slantface.
23. Gere et al., (2017)	Hungary	Insects as a substitute for meat	NS	NS
24. Gómez-Luciano, Vriesekoop, et al., (2019)	Dominican Republic and Spain	Insect proteins	NS	NS
25. Gómez-Luciano, de Aguiar, et al., (2019)	The United Kingdom Spain, Brazil Dominican Republic	Insect-based proteins	NS	NS
26. Hartmann & Siegrist, (2016)	Switzerland	Control group: Insects, deep-fried silkworms, deep-fried crickets<br>Experimental group: Tortilla chips (corn meal vs. cricket flour)	Visible and invisible	Silkworms, crickets
27. Hartmann et al., (2015)	Germany and China	Different food contexts (insects as a meat substitute, deep-fried silkworms, deep-fried crickets, drinks containing silkworm protein, cookies based on cricket flour and chocolate chip cookies based on cricket flour	Visible and invisible	Silkworms, crickets
28. House, (2016)	The Netherlands	Burgers, nuggets, schnitzel and pittige punten, all of which are made with vegetables and 13-15% ground-up buffalo worms, the larvae of the beetle	Invisible	Buffalo worms, larvae of the beetle
29. Iannuzzi et al., (2019)	Italy	Pizza with cricket flour and pizza with cricket flour and spirulina	Invisible	Cricket

30. Jensen & Lieberoth, (2019)	Denmark	Roasted mealworms, spring rolls sprinkled with visible roasted mealworms, spring rolls with mealworm flour, buttermilk soup sprinkled with roasted mealworms, buttermilk soup with processed mealworms	Visible and invisible.	Mealworms.
31. Kornher et al., (2019)	Germany	Beef burger patty fortified with insect flour	Invisible	NS
32. La Barbera et al., (2018)	Italy	Chocolate bar with peanuts enriched with protein from crickets	Invisible	Cricket
33. La Barbera et al., (2020)	Denmark and Italy	**S1** Mealworms, grasshoppers, ants<br>**S2** Insect products	NS	Mealworms, grasshoppers, and ants
34. Lammers et al., (2019)	Germany	Buffalo worms, buffalo worm burger	Visible and invisible	Buffalo worms
35. Laureati et al., (2016)	Italy	Biscuits made using insect flour, chocolate-coated grasshoppers, cereal bars containing insects, apple salad containing insects, tequila containing a larva, risotto containing maggots, maggot cheese, lollipops containing larvae	Visible and invisible	Grasshopper, larva, maggot
36. Le Goff & Delarue, (2017)	France	Potato chips claimed to be insect-based with 4 different flavours: strawberry, blackcurrant, chicken, barbecue	Invisible	NS
37. Lensvelt & Steenbekkers, (2014)	The Netherlands and Australia	Roasted crickets and a savory biscuit made with insect flour which contained a combination of ground crickets, mealworms, and pupae	Visible and invisible.	Crickets, mealworms, and pupae
38. Liu, Ai-Jun; Li, Jie; Gómez, (2020)	China	Edible insects	NS	NS
39. Lombardi et al., (2019)	Italy	Pasta, cookies, chocolate bars with non-visible mealworms and their conventional counterparts	Invisible	Mealworms
40. Mancini, Sogari, et al., (2019)	Italy	Bread with insect powder	Invisible	NS

41. Menozzi et al., (2017)	Italy	Chocolate chip cookie (containing 10% cricket flour)	Invisible	Cricket
42. Modlinska et al., (2020)	Poland	Cricket flour cookies, mealworm flour cupcakes, beetle flour date balls, cookies with crickets, cupcakes with particles of mealworm larvae, date balls with May beetle particles	Visible and invisible	Mealworms, cricket, beetle
43. Motoki et al., (2020)	Japan	**S1** Insect-based food<br>**S2** Insect-based food<br>**S3** Insect-based foods (mealworm burger, cricket chocolate bar)	Invisible	Mealworm, cricket
44. Myers & Pettigrew, (2018)	Western Australia	Entomophagy	NS	NS
45. Nyberg et al., (2020)	Sweden	Dried mealworms and crickets, bread with added cricket flour	Visible and invisible	Mealworms, crickets
46. Olum et al., (2020)	Uganda	Long-horned grasshoppers, flying African termites and the wingless red termites	Visible	Long-horned grasshoppers, flying African termites, and wingless red termites.
47. Onwezen et al., (2019)	The Netherlands	**S1** Grasshoppers, mealworms and beetles<br>**S2** Fresh insects, dried insects, fried insects, processed insects<br>**S3** Insect-based burger made from buffalo worms	**S1** Visible<br>**S2** Visible and invisible<br>**S3** Invisible	Grasshoppers, mealworms and beetles, buffalo worms
48. Orkusz et al., (2020)	Poland	Whole insects, bread with a 20% addition of powder from crickets	Visible and invisible	Crickets
49. Orsi et al., (2019)	Germany	Snack of buffalo worms, locusts, mealworms; granola mixed with buffalo worms; protein bar made with cricket powder; pasta made with buffalo worms; burger made with buffalo worms mixed with egg, soy and other ingredients	Visible and invisible	Buffalo worms, locusts, mealworms, crickets
50. Palmieri et al., (2019)	Italy	Insect-based food	NS	NS
51. Pambo et al., (2017)	Kenya	Cricket buns	Invisible	Cricket

52. Pambo, Okello, et al., (2018)	Kenya	Cricket-flour buns	Invisible	Crickets
53. Pascucci & De-Magistris, (2013)	The Netherlands	Insect-based product that looks like sushi	Visible and invisible.	NS
54. Payne, (2015)	Japan	Edible insects	NS	Wasp larvae, grasshopper
55. Petersen et al., (2020)	US	Chocolate brownie made with cricket powder	Invisible	Crickets
56. Piha et al., (2018)	(Northern and Central Europe- Finland, Sweden, Germany, and the Czech Republic)	Crunchy crickets for a snack with dipping sauce, a mix of ground ants and blueberries, cricket-rye snacks, giant mealworm wok, chicken-mealworm nuggets, crushed mealworms with chili	Visible and invisible	Cricket, ground ants, mealworms
57. Poortvliet et al., (2019)	The Netherlands	Insect burgers made from buffalo worms, mealworms, locusts; skewers insect cubes from buffalo worms, locusts	Invisible	Buffalo worms, mealworms, locusts
58. Powell et al., (2019)	UK	Insect-based burgers	Invisible	NS
59. Roma et al., (2020)	Italy	Cricket flour, cookies made from wheat and insect flour, cookies containing visible insects	Visible and invisible	Crickets
60. Ruby & Rozin, (2019)	US<br>India	Tacos with grasshoppers clearly displayed inside; a dosa (an Indian crepe made of rice and lentil flour), rolled up with a (non-visible, but verbally described) filling of potatoes and grasshoppers; six transparent lollipops half containing a mealworm and half containing a grasshopper	Visible and invisible	Mealworm and grasshopper
61. Rumpold & Langen, (2019)	Germany	Whole mealworms, locusts	Visible	Mealworms, locusts
62. Schösler et al., (2012)	The Netherlands	Variety of meat substitutes including pizza containing protein derived from insects, fried locusts with chocolate coating, locust salad, salad with fried mealworms	Visible and invisible	Mealworms, locusts

63. Schäufele et al., (2019)	Germany	Grasshoppers and mealworms (meatballs, whole, crushed)	Visible and invisible	Grasshoppers, mealworms
64. Schlup & Brunner, (2018)	Switzerland	Mealworms, locusts, caterpillars	Visible and invisible	Mealworms, locusts, caterpillars
65. Séré et al., (2018)	Burkina Faso (Sudanian zone)	Edible insects (Fried, roasted, ingredients)	Visible and invisible	Winged termites, caterpillars, grasshoppers, field cricket, beetles, palm weevil, Oryctes sp
66. (Simion et al., 2019)	Romania	Variety of insects including locusts, ants, and crickets.	NS	Variety but the most preferred are locusts and ants
67. Sogari et al., (2017)	Italy	Cookie made with cricket flour	Invisible	Crickets
68. Sogari et al., (2018)	Italy	Cricket-based jelly.	Visible and invisible	Crickets
69. Sogari et al., (2019)	Italy	Whole cricket in a jelly sweet and cricket flour in jelly sweet	Visible and invisible	Crickets
70. Sogari, (2015)	Italy	Crickets, honeycomb moths, wax moth larvae, and grasshoppers	Visible.	Crickets, honeycomb moths, wax moth larvae, and grasshoppers
71. Sogari, et al., (2019)	Australia (Sydney)	Edible insects, cricket flour or edible insects-filled chocolate bars	Visible and invisible	Cricket
72. Szendrő et al., (2020)	Hungary	Fried locusts and crickets, cakes contain insect flour	Visible and invisible	Locusts, crickets
73. Tan et al., (2015)	The Netherlands and Thailand	Ant larvae, big-butt ants, grasshoppers, giant water bugs, mopane worms, witchetty grubs, mealworms, bamboo worms, fried grasshoppers with chili and salt, mealworm muffins with chocolate pieces, cricket fritters with roasted peanuts, giant water bug chili paste, chocolate coated grasshoppers, Butter cookies with ground beetles	Visible and invisible	Ant larvae, big-butt ants, grasshoppers, giant water bugs, mopane worms, witchetty grubs, mealworms, bamboo worms, crickets, ground beetles
74. Tan et al., (2016)	The Netherlands	Burger labelled as mealworms (75% beef 25% mealworms)	Invisible	Mealworms

75. Tan, Tibboel, et al., (2017)	The Netherlands	Burger claimed to contain ground mealworms	Invisible	Mealworms
76. Tan, Verbaan, et al., (2017)	The Netherlands	Mealworm meatballs, mealworm drink	Invisible	Mealworms
77. Tuccillo et al., (2020)	Italy	Variety of insects and insects-based products such as crickets, grasshoppers, and three insects at the larval stage (bee, mealworm and silkworm larvae), cricket flour pasta, giant water bug chili paste, chocolate-covered grasshoppers, muffins with mealworms, fried rice with silkworms, focaccia bread with bits of dried crickets.	Visible and invisible	Cricket, giant water bugs, grasshoppers, three insects at the larval stage (bee, mealworm and silkworm larvae)
78. Van Thielen et al., (2019)	Belgium	Variety of products including energy shakes, energy bars, burgers, soup, sandwich spreads, snack	Invisible	Mealworms
79. Vanhonacker et al., (2013)	Belgium	Proteins from insects	NS	NS
80. Verbeke, (2015)	Belgium	Insects as a meat substitute	NS	NS
81. Verneau et al., (2016)	Denmark and Italy	Chocolate bar enriched with proteins from crickets	Invisible	Crickets
82. Verneau et al., (2020)	Italy and Denmark	Insect based food	NS	NS
83. Videbæk & Grunert, (2020)	Denmark	Variety of products e.g., baked, baguette baked with cricket flour, Purée of mushy peas and cricket flour, seasoned with garlic and lemon.	Visible and invisible	Crickets and mealworms
84. Wilkinson et al., (2018)	Australia	Flavored insects, chocolate-coated insects, biscuits made with insect flour, and a meal containing insects included as options, crickets, ants, witchetty grubs, mealworms, grasshoppers, scorpions, spiders, cockroaches.	Visible and invisible	Crickets, ants, witchetty grubs, mealworms, grasshoppers, scorpions, spiders, cockroaches

85. Woolf et al., (2019)	USA	Fried/grilled/toasted whole insects, chocolate coated insects, ground insects in sauces, chutneys, ground insects in burgers/nuggets/meatballs, bakery products, chips containing insect flour, rice/pasta enriched with insect flour, protein bars containing insect protein isolate	Visible and invisible	NS

NS: not specified.
